# Development of a decision aid to inform patients’ and families’ renal replacement therapy selection decisions

**DOI:** 10.1186/1472-6947-12-140

**Published:** 2012-12-01

**Authors:** Jessica M Ameling, Priscilla Auguste, Patti L Ephraim, LaPricia Lewis-Boyer, Nicole DePasquale, Raquel C Greer, Deidra C Crews, Neil R Powe, Hamid Rabb, L Ebony Boulware

**Affiliations:** 1 Welch Center for Prevention, Epidemiology and Clinical Research, Johns Hopkins Medical Institutions, Baltimore, Maryland, USA; 2 Division of General Internal Medicine, Johns Hopkins University School of Medicine, Baltimore, Maryland, USA; 3 Department of Epidemiology, Johns Hopkins Bloomberg School of Public Health, Baltimore, USA; 4 Division of Nephrology, Johns Hopkins Medical Institutions, Baltimore, Maryland, USA; 5 Department of Medicine, University of California San Francisco and San Francisco General Hospital, San Francisco, California, USA

**Keywords:** Kidney disease, Decision aid, Literacy, Numeracy, Cognitive function

## Abstract

**Background:**

Few educational resources have been developed to inform patients’ renal replacement therapy (RRT) selection decisions. Patients progressing toward end stage renal disease (ESRD) must decide among multiple treatment options with varying characteristics. Complex information about treatments must be adequately conveyed to patients with different educational backgrounds and informational needs. Decisions about treatment options also require family input, as families often participate in patients’ treatment and support patients’ decisions. We describe the development, design, and preliminary evaluation of an informational, evidence-based, and patient-and family-centered decision aid for patients with ESRD and varying levels of health literacy, health numeracy, and cognitive function.

**Methods:**

We designed a decision aid comprising a complementary video and informational handbook. We based our development process on data previously obtained from qualitative focus groups and systematic literature reviews. We simultaneously developed the video and handbook in “stages.” For the video, stages included (1) directed interviews with culturally appropriate patients and families and preliminary script development, (2) video production, and (3) screening the video with patients and their families. For the handbook, stages comprised (1) preliminary content design, (2) a mixed-methods pilot study among diverse patients to assess comprehension of handbook material, and (3) screening the handbook with patients and their families.

**Results:**

The video and handbook both addressed potential benefits and trade-offs of treatment selections. The 50-minute video consisted of demographically diverse patients and their families describing their positive and negative experiences with selecting a treatment option. The video also incorporated health professionals’ testimonials regarding various considerations that might influence patients’ and families’ treatment selections. The handbook was comprised of written words, pictures of patients and health care providers, and diagrams describing the findings and quality of scientific studies comparing treatments. The handbook text was written at a 4^th^ to 6^th^ grade reading level. Pilot study results demonstrated that a majority of patients could understand information presented in the handbook. Patient and families screening the nearly completed video and handbook reviewed the materials favorably.

**Conclusions:**

This rigorously designed decision aid may help patients and families make informed decisions about their treatment options for RRT that are well aligned with their values.

## Background

Patients whose advanced chronic kidney disease (CKD) is approaching end-stage renal disease (ESRD), or complete kidney failure, face complex medical decision-making regarding the type of medical therapy they wish to pursue. Choices for treating ESRD are numerous and include renal replacement therapies (RRTs) such as hemodialysis delivered in a dialysis center three times per week, home care dialysis (including peritoneal dialysis and home hemodialysis, which patients administer to themselves daily), and living or deceased kidney transplantation. Patients may also choose to forgo RRT and instead opt for conservative medical therapy (i.e., ongoing medical care with their nephrologists to optimize health without dialysis or kidney transplantation). Each treatment option has different advantages, limitations, and implications for survival, quality of life, financial stability, general health status, and how patients experience their daily lives [1-3]. For instance, home dialysis therapies require patients’ substantial self-care and may afford patients more control over their daily schedules, while in-center hemodialysis requires less self-management but more frequent interface with medical professionals and may offer patients less autonomy [4]. Patients’ families are also impacted by RRT decisions, as they often play substantial caregiver roles [5].

Despite patients’ critical need to understand the characteristics of various RRTs to inform their treatment decisions, many patients report having little to no general awareness of what types of treatment options exist for ESRD and many have little knowledge of treatment risks and benefits prior to their initiation of RRT [2,5-9]. Patients’ poor access to adequate education about treatments has been linked to their abrupt initiation of dialysis and their suboptimal access to other forms of RRT, [10,11] such as kidney transplantation, which is associated with improved clinical outcomes[6,12-14]. A substantial proportion of patients with ESRD are elderly and may have low educational attainment, poor health literacy and numeracy, and cognitive decline associated with their advancing kidney disease, heightening the challenges of educating patients about their RRT choices [15-18].

Comprehensive decision aids are needed to help patients with kidney disease and their families make informed RRT selection decisions aligned with their personal values. This paper describes our development of a patient-and-family centered decision aid to inform RRT selection decisions.

## Methods

### Overarching goals, decision Aid content, and approach to decision Aid design

#### Goals

We sought to develop a patient-and-family centered decision aid (handbook and video) to help patients and families choose among numerous RRT options. We sought to ensure that the decision aid could be understood by patients with a range of health literacy, health numeracy, and cognitive needs. We used the 2006 International Patient Decision Aids Standards (IPDAS) to guide our development process [19]. All aspects of the decision aid design, including the collection of primary patient and family data to inform development, were approved by the Johns Hopkins Medicine Institutional Review Board.

#### Decision aid content

We intended for the decision aid to address both positive and negative features of treatment options that patients (who had previously experienced treatments) and their family members felt others making RRT decisions should know about. We identified content to include via: (1) our own conduct of 20 focus groups of patients and families identifying information they felt should be included in decision aids guiding others’ RRT selections (described elsewhere) [5] as well as our selective review of qualitative studies published by other investigators, [2,3,20] and (2) our conduct of rigorous systematic literature reviews describing findings of scientific studies comparing outcomes among patients selecting different RRTs (described elsewhere) [21-24]. Our decision aid focused on seven key content areas or “concerns” about treatments, including how treatments might affect patients’: (1) morbidity and mortality (“health problems”), (2) autonomy (“doing things”), (3) treatment delivery (“how treatment works”), (4) symptoms (“symptoms”), (5) relationships (“relationships”), (6) psychological well being (“feelings”), and (7) finances (“money matters”) (Table 1).

**Table 1:** Topics addressed in the decision aid

**Concern**	**Factor**	**Video**	**Handbook**
**Morbidity/Mortality**	Living longer		
**(“Health Problems”)**	Infections	·	·
	Complications with surgery	·	·
	Making frequent trips to the doctor	·	
	Going to the hospital	·	
**Autonomy (“Doing Things”)**	Doing things I want to do when I want to do them	·	·
	Doing my usual activities	·	·
	Freedom and control over my life	·	·
	What I can eat or drink	·	
	Control over my treatment schedule	·	
	Going places by myself	·	
	My quality of life	·	·
	My social life	·	·
	Ability to do things in my free time	·	·
	How much free time I have	·	·
	How I feel about traveling	·	·
	Ability to go to work	·	·
	What I can do at work		·
	How I feel about my work	·	·
	My job and money	·	·
	How free I feel to do things		·
	Ability to do day-to-day tasks		·
	My ability to get around		·
	How I feel about getting around		·
	Caring for myself	·	·
**Treatment Delivery (“How Treatment Works”)**	Dialysis/transplant going as expected	·	
	Pills I have to take	·	
	Providing my own treatment	·	
	Ordering/storing supplies at home	·	
	Fistula or catheter problems	·	
	Finding a living donor	·	
	Surgery for fistulas or catheters	·	
	Blood tests, x-rays, and doctor visits	·	
**Symptoms(“Symptoms”)**	Feeling tired	·	·
	Thinking clearly	·	·
	My memory	·	·
	My attention	·	·
	How well I can learn		·
	Itching, cramping, or aching	·	·
	Gaining weight	·	
	Pain	·	·
	Joint pain		·
	How healthy my body feels		·
	My energy	·	·
	Cramps	·	·
	Stomach problems	·	·
	Bowel problems	·	·
	Cough		·
	Trouble breathing	·	·
	Skin problems		·
	Dry skin		·
	Changes in skin color		·
	How well I sleep		·
	How I feel about my looks	·	·
**Relationships (“Relationships”)**	Family and friends need to help	·	
	Making new friends	·	
	Having and enjoying sexual relations	·	·
	Sex drive	·	·
	Orgasm problems		·
	Pain with sex		·
	Erection problems (Men)	·	·
	Ejaculation problems (Men)		·
	Chances of having sex (Men)		·
	Trouble getting excited (Women)	·	·
	Vaginal dryness (Women)	·	·
**Psychological (“Feelings”)**	Feeling sad, anxious, or stressed out	·	·
	Chances of being depressed	·	·
	My nerves	·	·
	My well-being	·	·
	My emotions	·	·
	My mood	·	·
	How happy I am with my life	·	·
**Finance (“Money Matters”)**	Money spent from own pocket	·	

#### Approach to designing the decision aid

**Figure 1:**
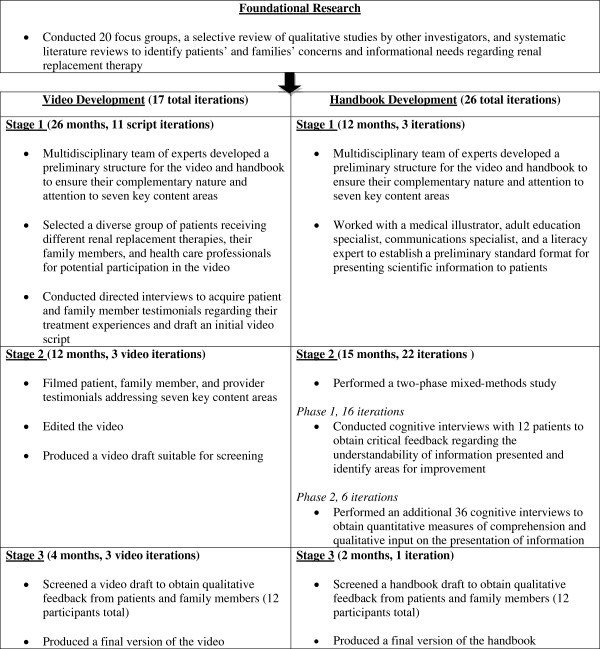
Decision aid development process.

We intended for the video and handbook to present complementary subjective (in video) and evidence-based (in handbook) information to a diverse English-speaking audience. We developed the video and handbook simultaneously in three “stages” (Figure 1). We engaged a multidisciplinary group of experts throughout the development process, which included clinical experts (physicians, social workers), an adult health education specialist, a patient advocacy specialist, a video scriptwriter, and a medical illustrator.

### Methods: developing the video

We intended to provide patients and their families with specific testimonials from others who had already made treatment decisions. We attempted to ensure these testimonials addressed as many of the seven previously identified “concerns” as possible. When patients or families did not address “concerns” in their testimonials, or if deemed appropriate, we incorporated testimonials from health care professionals who could provide additional views regarding common patient experiences relevant to the “concerns.”

#### Video development stage 1: selecting patients and families for the video, writing the script

##### Engaging a scriptwriter

We engaged a professional video scriptwriter with experience creating medical education videos. The scriptwriter reviewed findings from our foundational research and worked with our investigative team to develop questions designed to elicit positive and negative viewpoints from patients and families about their chosen RRTs.

Selecting patients and families and writing the script. We worked with local nephrologists in Baltimore, MD to identify a diverse group of patients who might potentially participate in the video. We asked nephrologists to select patients receiving different forms of RRT with specific demographic characteristics (e.g., minority female who received a transplant) to ensure diversity. The scriptwriter conducted 90 to 120 minute directed interviews with patients and family members and recorded the content of interviews for review.

The scriptwriter then drafted a script by incorporating views she obtained from patients and families. In our previously conducted focus groups, patients and families identified health care professionals they thought would be appropriate to address various “concerns.” For example, patients and family members felt physicians would be appropriate for discussions about the health risks and benefits associated with treatment selections, while social workers would be more appropriate for discussing financial considerations. Our investigative team reviewed the script and refined it to ensure all seven “concerns” were adequately addressed by either patients and families or health professionals (which included a transplant nephrologist, transplant social worker, general nephrologist, and dialysis social worker).

We also ensured that the video addressed complementary information to the handbook. Because the handbook summarized findings from scientific studies about the various RRT options (e.g., differences of health risks associated with different therapies), we did not include this type of quantitative information in the video (Table 1).

#### Video development stage 2: producing the video

##### Filming and producing the video

A professional video production crew filmed patients and family members in their homes during scheduled visits. To obtain testimonials on camera, an investigative team member interviewed them using the same questions the scriptwriter asked during initial directed interviews. The crew filmed health care personnel in a studio. An investigative team member asked health care providers questions to address issues that patient testimonials did not already address. After completing video filming and editing, we iteratively refined the video, focusing on decreasing length and improving clarity, consistency, and accuracy.

#### Video development stage 3: video screening and final edits

##### Screening groups

Prior to final editing, we recruited five patients receiving different RRTs from two local, academically affiliated nephrology practices in Baltimore, MD and seven of the patients’ family members to screen the video and provide their qualitative feedback during a two hour group meeting led by a trained moderator. The moderator elicited participants’ initial impressions of the video as well as feedback regarding length, amount of information presented, understandability, the balance in presentation of positive and negative aspects of the treatments, and areas for improvement. We audio-recorded and transcribed screening group discussions and we incorporated screening groups’ feedback in our final iterations.

### Methods: developing the handbook

The handbook complemented the video (Table 1) and summarized findings from scientific studies about differences in patient-reported and clinical outcomes among patients receiving different treatment options (in-center hemodialysis, peritoneal dialysis, kidney transplantation, and medical therapy with no dialysis or transplantation). We also provided information about the methodological quality of scientific studies. The handbook focused on the same seven “concerns” highlighted in the video.

#### Handbook development stage 1: preliminary handbook content design by our investigative team

We worked with specialists in medical illustration, adult education, health communications, and in adapting materials for low literacy readers to develop the handbook. We created several mock designs for presenting information before conducting the pilot study.

#### Handbook development stage 2: mixed-methods pilot study to guide iterative design

During early handbook development, we confronted three main challenges, including (1) presenting information about differences in multiple treatment types, (2) presenting information quantifying the magnitude and direction of scientific findings that did not incorporate risk probabilities and could not be described using standard methods (e.g. pictograms or bar graphs), [25] and (3) communicating information to patients about the quality of scientific evidence available to inform a decision. [26] To help address these challenges, we performed a pilot study among patients with kidney disease to obtain their input on the clarity of information we presented in various handbook iterations.

##### Pilot study goals, setting, and participants

In two study “phases,” we recruited a total of 48 English-speaking patients from two local nephrology practices in the Baltimore, MD metropolitan area to obtain qualitative feedback on handbook material (Phase 1 and 2) and quantitatively test patients’ comprehension of handbook material (Phase 2 only) during the iterative design process. For both phases, we asked nephrologists to identify patients either on dialysis or with progressive CKD (“Pre-ESRD,” defined as estimated glomerular filtration rate of less than 30 ml/min/1.73 m^2^) but not yet receiving dialysis or a kidney transplant. We then approached patients to assess their interest in participating in cognitive interviews.

##### Phase 1 cognitive interviews

During study Phase 1, we performed cognitive interviews with 12 patients with kidney disease to obtain feedback regarding the understandability of the information and ways to improve information presentation. We provided participants with a binder that included 8 to14 sample handbook pages. After participants reviewed each sample handbook page a trained interviewer asked six open-ended questions: 1) “Please tell me what you think the information on this page means,” 2) “What does (fill in topic name) mean to you?” 3) “We’re not sure if these are the best words to use, if you think there is a better way, we’d like to hear it,” 4) “Is any part of this page unclear?” 5) “Do you have any other suggestions?” and, 6) “On a scale of 1–5, with 1 being very hard to use and 5 being very easy to use, how would you score your ability to use this picture and the words to understand the information?” We audio-recorded and transcribed each interview. We reviewed transcriptions and made iterative changes to the handbook to address participants’ comments.

##### Phase 2 cognitive interviews and assessment of comprehension

During study Phase 2, we performed 36 additional cognitive interviews. As with Phase 1, we asked participants to provide qualitative feedback regarding the understandability and usability of information. In study Phase 2, we also assessed participants’ “gist” comprehension [27,28] of five key aspects of scientific evidence presented on each page, including whether they could understand: (1) the topic (“What issue are we talking about on this page?”), (2) the treatment modalities (“What treatments are being compared on this page?”), (3) the direction of differences in outcomes between RRTs being portrayed (“Which treatment is better?”), (4) the magnitude of difference in outcomes between RRTs (“How much better or worse is one treatment versus the other?”), and (5) the research quality of scientific studies to inform that aspect of the decision (“Does the page tell people how good the information is?”).

We asked participants to view 7 to 9 different sample handbook pages displaying similar types of information (including information addressing five key aspects of scientific evidence: topic, treatment modalities being compared, direction of differences between treatments, magnitude of differences, and research quality). We assessed participants’ gist comprehension of each key aspect on each page. Two independent investigators reviewed transcripts of each audio-recorded interview to assess participants’ gist comprehension and scored participants’ responses as correct or incorrect. A third research team member adjudicated any discrepancies among the two reviewers by also reviewing the transcripts.

For each key aspect of scientific evidence, we calculated the number of pages for which participants correctly demonstrated comprehension. For example, if a participant demonstrated correct comprehension of the treatments displayed on 7 of 9 pages, we assessed that participant as having correct comprehension of this element 78% of the time. We then summarized the total group’s comprehension of each element by calculating the median (range) of all participants’ percentages indicating correct comprehension of that element. We also assessed patient participants’ demographic characteristics; health literacy (Rapid Estimate of Adult Literacy in Medicine) [29]; numeracy level (Risk Numeracy) [30]; cognitive function (Trails B) [31]; and knowledge and awareness of kidney disease [7].

#### Handbook development stage 3: handbook screening and final edits

Patients and family members who participated in the video screening (described above under “Video Development, Stage 3”) also reviewed the handbook and provided opinions to the same open-ended questions.

## Results

**Figure 2:**
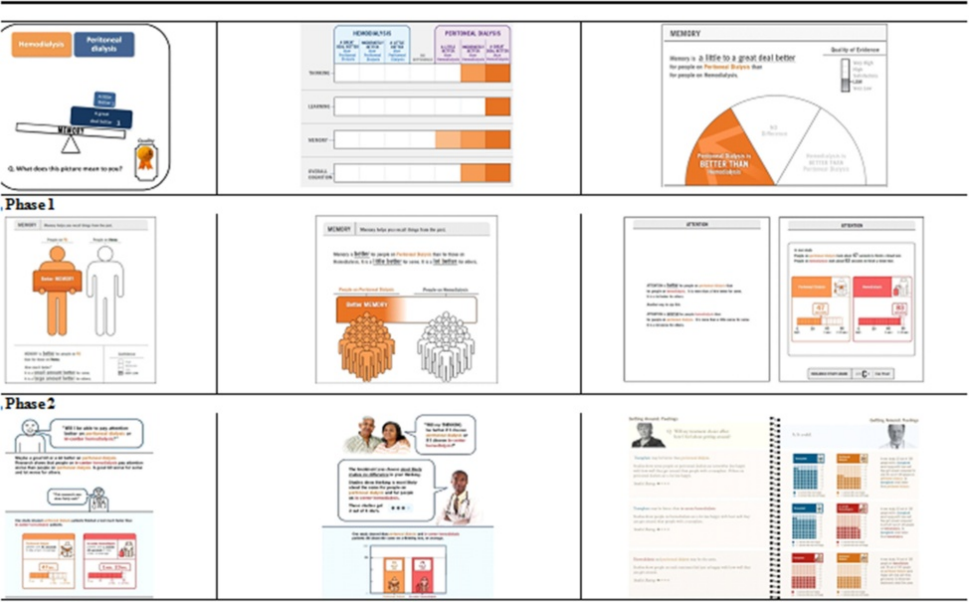
Representative sample iterations from stages 1 and 2 of the handbook development process.

Feedback we received during each phase resulted in numerous iterative refinements of both the video and the handbook. We iteratively refined the video 17 times (11 script iterations in Stage 1, three initial video iterations in Stage 2, and three final film iterations in Stage 3). We iteratively refined the handbook 26 times (three iterations in Stage 1, 22 iterations in Stage 2, and one final iteration in Stage 3). Examples of preliminary handbook presentations, reflecting major iterative design approaches from Stages 1 and 2, are presented in Figure 2.

### Results: findings from mixed methods pilot study

Of the 12 participants in pilot study Phase 1, 67% were male, 92% were African American, and 75% had at most a high school degree or GED. The majority of participants were on hemodialysis (n=9, 75%), while the remaining participants were classified as pre-ESRD (n=3, 25%). Of the 36 participants in Phase 2, 44% were male, 83% were African American, and 47% had at most a high school degree or GED. Fifty-four percent of phase 2 participants were on hemodialysis, 33% were considered pre-ESRD, 29% on peritoneal dialysis, and 17% on home hemodialysis. Participants in both phases were diverse with respect to their knowledge of kidney disease, literacy, numeracy, and cognitive function (Table 2).

**Table 2:** Participant characteristics for handbook development Stage 2 (Phases 1 and 2)

**Patient characteristics, n(%)**	**All participants (Phases 1 & 2)**	**Phase 1 participants**	**Phase 2 participants**
	**(n=48)**	**(n=12)**	**(n=36)**
**Age**			
Mean [Range]	59 [33–85]	58 [46–71]	60 [33–85]
**Gender**			
Male	24 [50%]	8 [67%]	16 [44%]
**Race/Ethnicity**			
African American	41 [85%]	11 [92%]	30 [83%]
non-African American	7 [15%]	1 [8%]	6 [17%]
**Education**			
Less than a high school degree	9 [19%]	4 [33%]	5 [14%]
High school degree/GED	17 [35%]	5 [42%]	12 [33%]
Some college	14 [29%]	3 [25%]	11 [31%]
Bachelor’s degree	6 [13%]	0 [0%]	6 [17%]
Graduate or professional school	2 [4%]	0 [0%]	2 [5%]
**Treatment Group**			
Pre-ESRD	15 [31%]	3 [25%]	12 [33%]
Hemodialysis	22 [46%]	9 [75%]	13 [54%]
Home Hemodialysis	4 [8%]	---	4 [17%]
Peritoneal Dialysis	7 [15%]	---	7 [29%]
**Knowledge of Kidney Disease**^**§**^			
Extensive knowledge	4 [8%]	1 [8%]	3 [8%]
A great deal of knowledge	20 [42%]	4 [33%]	16 [44%]
Some knowledge	18 [38%]	7 [59%]	11 [31%]
Limited to no knowledge	5 [10%]	0 [0%]	5 [14%]
Don’t know	1 [2%]	0 [0%]	1 [3%]
**Literacy**			
3^rd^ grade and below	5 [10%]	1 [8%]	5 [14%]
4th to 6^th^ grade	4 [8%]	2 [17%]	2 [5%]
7^th^ to 8^th^ grade	14 [29%]	6 [50%]	8 [22%]
9^th^ grade and above	25 [52%]	3 [25%]	22 [61%]
**Numeracy**			
Risk numerate^€^	14 [30%]	3 [25%]	11 [31%]
**Cognition**^¥^			
Cognitive impairment	10 [21%]	3 [25%]	7 [19%]

^**§**^ Based on self-report.

^¥^ Time of 273 seconds or longer indicates a cognitive deficiency, 2 participants missing data from Phase 2.

^€^Considered risk numerate if 3 of 3 risk numeracy questions answered correctly, 1 participant missing from Phase 2.

Qualitative feedback obtained during cognitive interviews (during Phase 1 and Phase 2) focused on three key areas: (1) difficulties identifying the multiple treatment types presented in the handbook, (2) the handbook containing an intimidating amount of complex scientific information, and (3) their desires to have numerical or quantitative information from scientific studies presented in a way that was clear and understandable. Through a total of 22 iterations (16 iterations in Phase 1 and six iterations in Phase 2) we addressed these concerns through numerous adjustments (including the use of color coding to consistently identify RRT options when discussed throughout the handbook, providing explanatory guides for how to read pages, eliminating the use of abbreviations, and supplementing graphical numerical presentations with text written at a 4^th^ to 6^th^ grade reading level) (Table 3) .

**Table 3:** Qualitative feedback from handbook development Stage 2 (Phases 1 and 2)

**Patient concerns**	**Representative quotes**	**Specific challenges encountered**	**Solutions**
Patients did not know their treatment options	“First, can you explain the two treatments? What is the difference between those two treatments?”	· Defining patients’ various treatment options	· Added a treatment definition page (“What are the Treatments?”)
			· Replaced all abbreviations with actual treatment names
		· Making complex medical terminology memorable	· Color-coded each treatment option
			· Associated each treatment with its own icon
Intimidating amount of complex information	“And I just feel like this is so much information that's written that is not going to be taken in.”	· Translating research evidence into plain language	· Developed a question and answer format in plain language
			· Revised the language in the to achieve a fourth grade reading level
			· Created a new section (“What is on Each Page?”) to introduce and define research quality
		· Communicating research quality	· Used pictures of “real” doctors and patients diverse in age, sex, and gender
			· Placed tabs throughout the handbook to divide it into smaller sections
		· Making the handbook user-friendly	· Added an interactive value clarification exercise (“How Do I Choose a Treatment?”)
Understanding numerical information or statistical concepts	“I don’t want these chances or things…it’s real confusing. I want to know the facts.”	· Presenting graphical illustrations of data	· Used graphical presentations patients responded to most positively
			· Supplemented graphical presentations with text to reiterate the intended message
			· Adopted a double page spread format to appeal to a diverse group of readers
		· Using both positive and negative framing of statistical information	· Used an example study to anchor each head-to-head treatment comparison
		· Explaining effect size	· Modified effect size terminology from a “small/medium/large amount” to “a little/somewhat/ a lot better”

During study Phase 2, we measured gist comprehension of materials among a total of 36 participants. Twenty-four (24) participants provided feedback on iteration 20 of the handbook and 12 participants provided feedback on iteration 21 of the handbook. Among all items viewed by participants, participants demonstrated median gist comprehension of 78% (range: 15%–100%) in iteration 20. Participants’ median overall gist comprehension rose to a median of 91% (range: 9%–100%) in iteration 21. Participants demonstrated greatest gist comprehension on aspects of pages reflecting the topic being discussed, the treatment types (modalities) being compared, and the direction of scientific findings discussing treatment differences (i.e., they could correctly identify one treatment was better than the other). Participants demonstrated less gist comprehension regarding the magnitude of treatment differences (i.e., they had difficulty understanding *how much* better one treatment was than another) and information about the research study quality. However, gist comprehension scores improved in these areas between iterations 20 and 21 (Table 4).

**Table 4:** Median percentage** of times participants correctly comprehended key aspects of scientific evidence during pilot testing (handbook development Stage 2, Phase 2)

Key aspects of scientific evidence^*^	**Iteration 20**	**Iteration 21**
	**(n=24)**	**(n=12)**
Topic	100% [22%–100%]	100% [0%–100%]
Treatment Modality	100% [33%–100%]	100% [29%–100%]
Direction of Difference	89% [22%–100%]	86% [14%–100%]
Magnitude of Difference	71% [0%–100%]	100% [0%–100%]
Research Quality	56% [0%–100%]	100% [0%–100%]
**Total: All Items**	78% [16%–100%]	91% [9%–100%]

*Correct identification of five key items shown as a median percentage. followed by the respective range.

**Ranges in the table represent [minimum-maximum] values.

### Results: feedback from decision Aid screening

Five patients and seven family members participated in two different screening groups. Patient participants were 80% female, 60% African American, and 60% had at least two years of college education. All family member participants were female, 71% were African American, and 43% had at least two years of college education.

Patients and family members provided mostly positive feedback after screening both the video and handbook. (Table 5) Participants often commented that they liked the organization of the video and the breadth of topics it covered. Participants also commented on the complementary nature of the two materials. Screening group participants constructively criticized the large amount of information presented in the handbook. To address this concern, we created a mini-book entitled, “All of the Facts,” to accompany the handbook, which provides readers with a brief summary of key information presented in the longer handbook.

**Table 5:** Positive and constructive feedback obtained from screening the decision aid

**Reactions**	**Video**		**Handbook**	
	**Positive feedback**	**Constructive feedback**	**Positive feedback**	**Constructive feedback**
Overall Impression	“… if it was something like that available…when I started dialysis it would have really been welcome because information was scattered…And this, I like the way it’s organized, the way it’s broken down… I think it’s touched just about on everything that your initial concerns would be.”	“What I did have an issue with was the sequence of, I liked the way everything was broken down but I think it should have been sequenced differently starting with dialysis at the center, because they said that was what’s most frequently done, most frequently used.”	“I really do love this book. It does give a whole lot of information for someone who’s just starting out. You know, they really need all of the information they can-that they can get. And I love the fact that they would have a DVD to go along with it.”	“A lot of times if a person is just starting dialysis, you know, you got the sluggishness in your brain, your memory, and your attention. And it might be a little hard to focus on this as opposed to seeing a film, hearing people’s testimonies, you know, I think that would settle a little better than, you know, trying to absorb all of this.”
Length	“But I didn’t mind the length of it because it gave, you know, information that you need to know.”	“I thought it was too long. But as it went on we could see how important it was for it to be that long and I think if I was in that situation, it might not have been that long but since it wasn’t my issue, too long.”	“When I first saw it, it felt overwhelming but it was broken down so well that I can just go to the parts that I think are relevant to myself or that I want to see that day… it's separated so well that I can just go and look at it.”	“I mean, the inside of it, the content is good. I like the graphics in it and the charts…but I guess if it was some kind of way you could kind of condense it because they’re probably already getting a whole lot of other information at the same time to have to carry a big book.”
Amount of Information	“I think it's very informative and has just the right amount of information.”	Constructive feedback not available	“I think this is a terrific reinforcing publication plus when you’re watching a film you can only remember so much whereas you’re going to go back here and you’re going to see all of it in the different forms… Reading and writing solidify your thinking…I like it.”	“It could be very daunting just even looking at it. But if you have a video hopefully it will pique your interest enough to be able to go to the individual area that you’re concerned with and get that information that you need.”
Easy to Understand	“I did learn a lot of information from watching the movie. Even having some relatives that had kidney issues, this still provided more information.”	Constructive feedback not available	“It’s doesn’t go to a junior high school level so that means that everybody can understand it. So yeah I think it's clear.”	“The spoken word, a lot of times, is much easier. I think the combination is a great vehicle, I do. But I think with this, you know, that’s one of the things that I’d be concerned about. ‘Cause someone with a fifth grade reading level wouldn’t really be able to understand everything.”
Balanced Presentation of Treatment Options	“I don’t think they leaned anywhere. In fact, they kept saying basically it was your options to select or decide which way you want to go and you even had an option to rescind that and go in another direction.”	Constructive feedback not available	“Yeah it's pretty well balanced because it has the section for each treatment, you know, each mode in there so it's balanced.”	Constructive feedback not available
Areas of Improvement	Not applicable	“I think the one improvement that I would suggest is that [they put] a little bit more emphasis in each type of treatment about the emotional impact on not just you but also your family and your support system.”	Not applicable	“I think the best part and most useful part for most of the population is the all the facts part. It’s nice how they’ve compared the transplant, which is the ideal, to the other treatments. It might be helpful if there were a foldout where all the treatments were laid out like this to just read across.”

### Results: final decision Aid

The final decision aid consists of a 50-minute video and a comprehensive, 159-page handbook accompanied by a 14-page mini-book (“All of the Facts”) which summarizes the key information in the handbook. Together, the video and handbook satisfied 92% of all quality criteria outlined in the three subject areas designated by IPDAS (satisfied 96% or 22 of 23 content items, 87% or 20 of 23 development process items, and 100% or 6 of 6 effectiveness items) (Additional file 1).

The video provides testimonials from minority and non-minority (male and female) patients, their family members, and health care professionals (physicians and social workers) about “concerns” patients and their families might have which could influence their RRT selection decisions. These “concerns” correspond to the seven key content areas patients and their families identified as important to include in the decision aid during our foundational research studies. Patients and family members receiving different RRTs share subjective testimonials describing their positive and negative experiences with these “concerns” in the video. Health care providers discuss additional information regarding “concerns” to balance patient and family views and to contribute additional information.

**Figure 3:**
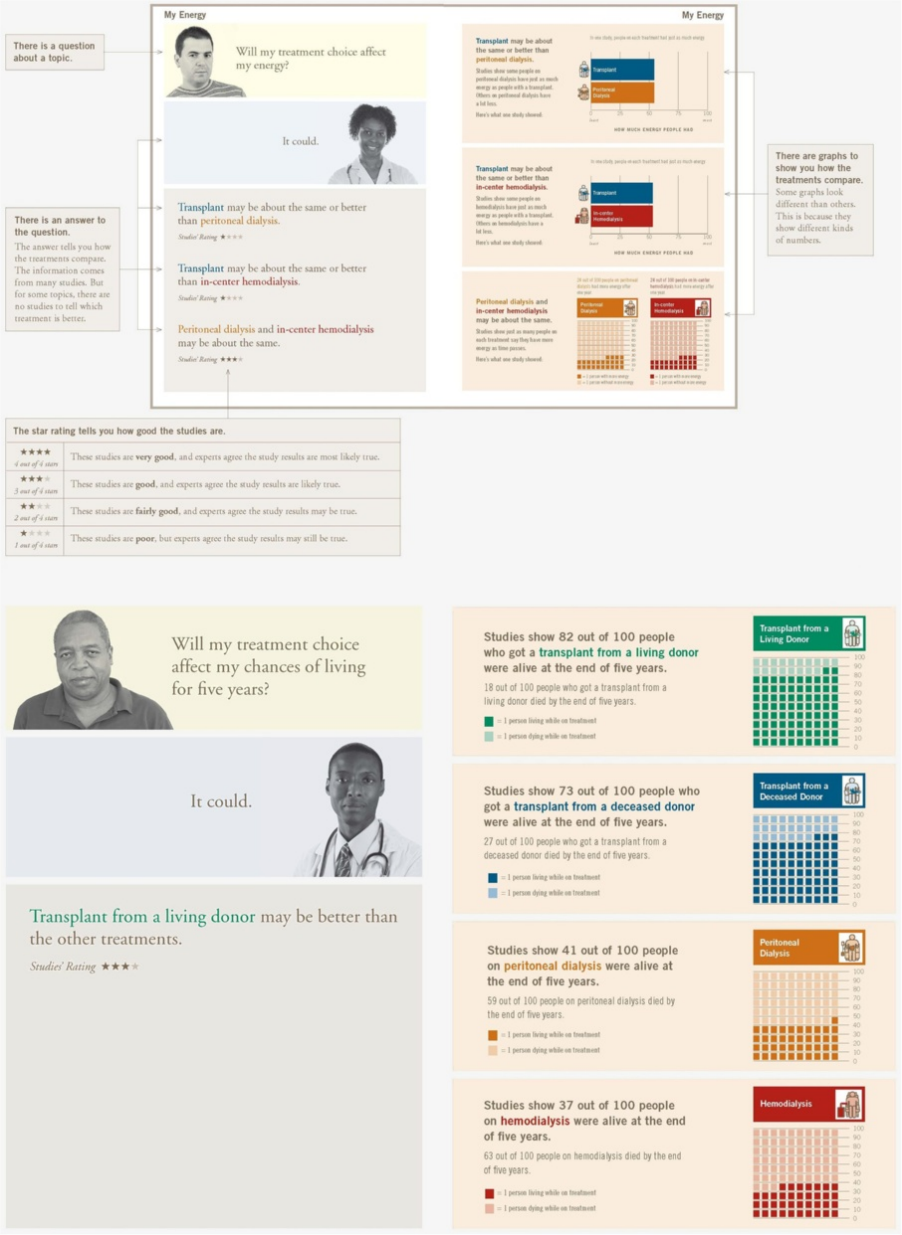
Sample pages from final handbook.

The handbook provides evidence-based information regarding risks and benefits of different treatment options of peritoneal dialysis, in-center hemodialysis, home hemodialysis, kidney transplant, and conservative management (treatment with no transplant or dialysis) as they pertain to these same patient concerns. The introduction includes a section that defines kidney disease (“What Is Kidney Disease?”), describes and color codes the different treatment options (“What are the Treatments?”), presents a value clarification exercise to help patients determine which treatment is best for them (“How Do I Choose a Treatment?”), and orients the reader to the page layout (“What is on Each Page?”). Subsequent sections of the handbook describe summaries of scientific studies relevant to each “concern.” Handbook pages are 8.5” by 11” and oriented toward a 4^th^ to 6^th^ grade reading level. The handbook delivers content on a double-page spread layout to appeal to diverse learning preferences: the left-hand page includes brief statements summarizing key messages from scientific studies while each right-hand page presents a more detailed summary of study findings and a graphical presentation of data (Figure 3).

## Discussion

Patients need high quality decision aids to help guide them through the decision-making process about RRT selection. To our knowledge, rigorous and systematic approaches to developing decision aids for patients with CKD have not been previously undertaken. We developed our decision aid to accommodate patients with varying literacy, numeracy, and cognitive needs. The final decision aid achieved the vast majority of IPDAS criteria for engaging patients in informed decision-making. Our refinement process incorporated 11 video script iterations and 26 handbook iterations, reflecting the extensive effort we undertook to develop materials that addressed patients’ self-reported informational needs. Initial screening of our decision aid by patients and their families indicates that these materials are likely to be well received. Pilot testing in our mixed-methods study suggested patients found the final handbook understandable. However, further testing is still needed to determine how effective or useful the decision aid is for ESRD patients’ treatment decisions.

Our development process and findings highlight some of the challenges of developing comprehensive materials for a diverse population that address the positive and negative features of various RRT options. First, we based our content on rigorous foundational studies (20 focus groups and systematic literature reviews), which required substantial time and effort to conduct. Translating our findings from these studies to inform decision aid development also proved challenging as we sought to address multiple concerns identified by patients and invested additional significant time and effort performing the mixed-methods pilot study to identify ways to convey scientific information in a way that can be easily interpreted by the vast majority of patients. Further, factors such as the layout of information and the inclusion of figures are known to be potential sources of bias in decision making. For instance, the final handbook (150 pages) and video (50 minutes) were lengthy. Although we did create a shorter “All the Facts” 14-page handbook in response to participants’ comments during the screening sessions, we did not formally compare how participants viewed this distilled version of information compared to the complete handbook and video. Further testing will help clarify the optimum format for information. We are currently studying these materials in a randomized controlled trial to examine their influence on treatment decisions in African Americans [32], and we are designing additional studies to help further refine the decision aid.

Incorporating subjective testimonials about treatment could introduce bias as patients may identify with video participants rather than synthesize information on preference sensitive topics. [33-35] Nonetheless, treatments for ESRD are quite complex, requiring many practical considerations that significantly alter patients’ daily lives. We therefore felt it was very important to show viewers examples of patients and families undergoing these treatments. For instance, our video shows a patient who receives home hemodialysis inserting a needle into his dialysis fistula. It shows another patient receiving hemodialysis in a treatment unit, and yet another patient administering peritoneal dialysis in her home with help from her spouse. The video depicts patients storing boxes of treatment supplies, handling medications, and traveling to the dialysis treatment facility. Without direct visualization, many patients considering these treatments may not fully comprehend the significant differences between therapies. Future studies will be needed to assess whether viewers of our video felt compelled to choose a treatment because of their subjective identification with video participants.

Some additional limitations of our process are important to consider. Although our team comprised experts from multiple disciplines, it did not include a decision scientist, which may have influenced our development approach. We did, however, refer to the decision sciences literature extensively as well as the IPDAS criteria throughout the development process [19,25-28,36-40]. We also developed our decision aid with input from a largely minority (African American) population of patients with kidney disease living in the Baltimore, MD metropolitan area. It is possible patients with different characteristics could have different viewpoints about RRT which might have altered our development process. Further, we developed our decision aid for English-speaking patients. Materials may require significant adaptation to accommodate reading and cultural needs of non-English speakers.

## Conclusion

We developed a decision aid to help patients with kidney disease and their families make informed decisions about RRT selections aligned with their values. While our development process helped to ensure completeness and readability of our decision aid, its effectiveness on aiding patients’ treatment decisions warrants further study.

## Abbreviations

CKD: Chronic kidney disease; ESRD: End-stage renal disease; RRT: Renal replacement therapy; IPDAS: International patient decision aid standards.

## Competing interests

The authors declare that they have no competing interests.

## Authors’ contributions

JA made substantial contributions to conception and design, acquisition of data, analysis and interpretation of data, was involved in drafting the manuscript or revising it critically for important intellectual content, and gave final approval of the version to be published. PA made substantial contributions to conception and design, acquisition of data, analysis and interpretation of data and gave final approval of the version to be published. PE made substantial contributions to conception and design, acquisition of data, analysis and interpretation of data and gave final approval of the version to be published. LLB made substantial contributions to conception and design, acquisition of data, analysis and interpretation of data and gave final approval of the version to be published. ND made substantial contributions to conception and design, analysis and interpretation of data, was involved in drafting the manuscript or revising it critically for important intellectual content, and gave final approval of the version to be published. RG made substantial contributions to conception and design, analysis and interpretation of data and gave final approval of the version to be published. DC made substantial contributions to conception and design, analysis and interpretation of data and gave final approval of the version to be published. NP made substantial contributions to conception and design, acquisition of data, analysis and interpretation of data and gave final approval of the version to be published. HR made substantial contributions to conception and design, acquisition of data, analysis and interpretation of data and gave final approval of the version to be published. LEB led the conception and design, acquisition of data, analysis and interpretation of data, and was involved in drafting the manuscript as well as revising it critically for important intellectual content. LEB also gave final approval of the version to be published. All authors read and approved the final manuscript.

## Pre-publication history

The pre-publication history for this paper can be accessed here:

http://www.biomedcentral.com/1472-6947/12/140/prepub

## Supplementary Material

Additional file 1 IPDAS criteria met by video and handbook decision aid. Click here for file
