# Supplementary material for: Development of a decision aid to inform patients’ and families’ renal replacement therapy selection decisions
Source: BMC Med Inform Decis Mak. 2012 Dec 1;12:140. doi: 10.1186/1472-6947-12-140 (PMC3560257; doi:10.1186/1472-6947-12-140)
Supplement: Additional file 1 — IPDAS criteria met by video and handbook decision aid. [file 1472-6947-12-140-S1.pdf]

Additional File 1. IPDAS criteria met by video and handbook decision aid

Criteria	Handbook	Video
**Content: Does the patient decision aid…**		
**Provide information about options in sufficient detail or decision making?**		
• Describe the health condition	•	•
• List the options	•	•
• List the option of doing nothing	•	•
• Describe the natural course without options	•	•
• Describe procedures	•	•
• Describe positive features (benefits)	•	•
• Describe negative features of options (harms/side effects/ disadvantages)	•	•
• Include chances of positive/negative outcomes	•	•
**Present probabilities of outcomes in an unbiased and understandable way?**		
• Use event rates specifying the population and time period	•	Not applicable
• Compare outcome probabilities using the same denominator, time period, scale	•	Not applicable
• Describe uncertainty around probabilities	•	Not applicable
• Use visual diagrams	•	Not applicable
• Use multiple methods to view probabilities (words, numbers, diagrams)	•	Not applicable
• Allows the patient to select a way of viewing probabilities (words, numbers, diagrams)	•	Not applicable
• Allow patient to view probabilities based on their own situation (e.g. age)	Not met	Not applicable
• Place probabilities in context of other events	•	Not applicable
• Use both positive and negative frames (e.g. showing both survival and death rates)	•	Not applicable
**Include methods for clarifying and expressing patients' values?**		
• Describe the procedures and outcomes to help patients imagine what it is like to experience their physical, emotional, social effects	•	•
• Ask patients to consider which positive and negative features matter most	•	•
• Suggest ways for patients to share what matters most with others	•	•
**Include structured guidance in deliberation and communication?**		
• Provide steps to make a decision	•	•

Criteria	Handbook	Video
• Suggest ways to talk about the decision with a health professional	•	•
• Include tools (worksheet, question list) to discuss options with others	•	•
**Development Process: Does the patient decision aid…**		
**Present information in a balanced manner?**		
• Able to compare positive/negative features of options	•	•
• Shows negative/positive features with equal detail (fonts, order, display of statistics)	•	•
**Have a systematic development process?**		
• Include developers' credentials/qualifications	•	•
• Finds out what users (patients, practitioners) need to discuss options	•	•
• Has peer review by patient/professional experts not involved in development and field testing	•	•
• Is field tested with users (patients facing the decision; practitioners presenting options)	•	•
**The field tests with users (patients, practitioners) show the patient decision aid is:**		
• Acceptable	•	•
• Balanced for undecided patients	•	•
• Understood by those with limited reading skills	•	•
**Use up to date scientific evidence that is cited in a reference section or technical document?**		
• Provides references to evidence used	Not met	Not met
• Report steps to find, appraise, summarize evidence	•	•
• Report date of last update	•	•
• Report how often patient decision aid is updated	•	•
• Describe quality of scientific evidence (including lack of evidence)	•	Not applicable
• Uses evidence from studies of patients similar to those of target audience	•	•
**Disclose conflicts of interest?**		
• Report source of funding to develop and distribute the patient decision aid	•	•
• Report whether authors or their affiliations stand to gain or lose by choices patients make after using the patient decision aid	Not met	Not met
**Use plain language?**		
• Is written at a level that can be understood by the majority of patients in the target group	•	•
• Is written at a grade 8 equivalent level or less according to readability score (SMOG or FRY)	•	•

Criteria	Handbook	Video
• Provides ways to help patients understand information other than reading (audio, video, in-person discussion)	•	•
**Meet additional criteria if stories are used in the patient decision aid?**		
• Use stories that represent a range of positive and negative experiences	Not applicable	•
• Reports if there was a financial or other reason why patients decided to share their story	Not applicable	•
• State in an accessible document that the patient gave informed consent to use their stories	Not applicable	Not met
**Effectiveness: Does the patient decision aid ensure decision making is informed and values based?**		
**Decision processes leading to decision quality. The patient decision aid helps patients to…**		
• Recognize a decision needs to be made	•	•
• Know options and their features	•	•
• Understand that values affect decision	•	•
• Be clear about option features that matter most	•	•
• Discuss values with their practitioner	•	•
• Become involved in preferred ways	•	•
**Decision quality. The patient decision aid…**		
• Improves the match between the chosen option and the features that matter most to the informed patient	Ongoing study	Ongoing study

*International Patient Decision Aids Standards (IPDAS) Collaboration. Developing a quality criteria framework for patient decision aids: online international Delphi consensus process. *BMJ*. 2007; 333(7565):417.
